# Effect and Threshold of Endoscopic Findings for CRS Control Status and Long‐Term Outcome Prediction

**DOI:** 10.1002/alr.70083

**Published:** 2025-12-19

**Authors:** Steven Chun‐Kang Liao, Aditi Agarwal, Junqin Bai, Zhidi Luo, Siyuan Dong, Regan L. Harmon, Brooke N. Gleason, Julia Huang, David B. Conley, Kevin C. Welch, Stephanie Shintani‐Smith, Robert C. Kern, Atsushi Kato, Lutfiyya N. Muhammad, Bruce K. Tan

**Affiliations:** ^1^ Department of Otolaryngology Northwestern University Feinberg School of Medicine Chicago Illinois USA; ^2^ Department of Otolaryngology National Taiwan University Hospital Yunlin Branch Yunlin Taiwan; ^3^ Department of Preventive Medicine (Biostatistics) Northwestern University Feinberg School of Medicine Chicago Illinois USA; ^4^ Division of Allergy and Immunology Department of Medicine Northwestern University Feinberg School of Medicine Chicago Illinois USA

**Keywords:** Chronic Sinusitis, Disease Control, Endoscopy

## Abstract

**Background:**

EPOS 2020 defined chronic rhinosinusitis (CRS) disease control using patient symptoms and medication usage but endoscopic findings were considered optional. The effect of adding endoscopic features, an appropriate threshold, and their association with present or future symptom control have not been studied.

**Methods:**

A prospective cohort study of 188 adult CRS patients undergoing bilateral endoscopic sinus surgery from 2017 to 2023 was conducted. Patients were assessed at 6–12 months (V1) and 18–60 months (V2) postoperatively. Individual patient symptoms from the SNOT‐22, endoscopic findings (modified Lund–Kennedy [MLK] score), and medication usage were recorded. CRS control status was classified as controlled or suboptimal control (EPOS partly/un‐controlled) based on EPOS 2020 criteria without endoscopic features. The predictive role of endoscopic findings was analyzed.

**Results:**

Endoscopic findings were weakly associated with concurrent control status but the total MLK (V1 AUC = 0.631, *p* < 0.001; V2 AUC = 0.620, *p* < 0.05) outperformed any individual MLK component in strength of association. Adding MLK to V1 control status using a threshold of MLK ≥ 3 marginally improved prediction accuracy for V2 control compared to V1 control status defined without endoscopic features (AUC = 0.744 vs. 0.721, respectively, both *p* < 0.001).

**Conclusion:**

While endoscopic findings are only weakly associated with concurrent control status, their addition enhances prediction of subsequent V2 CRS outcomes. Our study provides the first real‐world evidence supporting the value of endoscopic findings as predictors for CRS disease progression, with an MLK threshold ≥ 3 showing moderate predictive accuracy for future maintenance of control.

## 1 Introduction

Chronic rhinosinusitis (CRS) is a chronic inflammatory disease with definitional symptomatic and either endoscopic or radiographic findings. Treatments like endoscopic sinus surgery (ESS) and biologics have well‐characterized effects on symptoms and endoscopic findings, leading to improvements in disease control. However, disease definitions incorporating control have only recently been developed.

The EPOS 2020 [1] provided definitions of control status based on three levels of clinical control: controlled, partly controlled, and uncontrolled. These classifications are determined based on individual clinical symptoms, including nasal blockage, rhinorrhea/postnasal drip, facial pain/pressure, smell disturbances, sleep disturbance, and fatigue as well as rescue treatment usage (within the past 6 months). The presence of at least one of these items was defined as partly controlled, while the presence of three or more of these items was classified as uncontrolled [1, 2]. The presence of “diseased mucosa” on nasal endoscopy (NE) was an optional item in the definition of control and, if present, comprised a single item [1].

The definition and threshold of “diseased mucosa” has not been quantitatively evaluated and whether its addition improves the stability and prognostic value of control has not been explored. Traditionally, endoscopy evaluates qualitatively different items with commonly reported items including nasal polyp size, presence of edema, and the quality of mucus frequently evaluated in guidelines and clinical scoring systems [1]. Commonly utilized endoscopic scoring systems include the nasal polyp score (NPS) that scores only polyp size [3] and the modified Lund–Kennedy (MLK) score that evaluates polyp size, discharge, and edema separately [1, 4].

The added value of incorporating endoscopic findings into CRS control assessments remains uncertain. Multiple studies have shown that endoscopic scores correlate poorly with patients’ overall disease‐control status [5, 6, 7]. Venn et al. reported that including endoscopic findings in the EPOS 2012 criteria reclassified only four of 81 (4.9%) patients [8]. Despite this, expert opinion continues to recommend using endoscopic findings as a tool for evaluating CRS control and medication adjustment method [3], and it remains an important component of the comprehensive evaluation of CRS for rhinologists [9]. While this consensus has not provided an evidence‐based threshold for assessment, it has suggested using an NPS ≥ 3 or an MLK score ≥ 4 may serve as thresholds for considering CRS treatment escalation [3]. In a recent analysis, we studied the implications of radiographic outcomes after ESS and found that achievement of radiographic and symptomatic normalization measured using patient‐reported outcomes was significantly predictive of maintaining this status but have not studied implications and thresholds in the context of EPOS control status [10, 11]. This study aims to investigate the nature and relationship of qualitative endoscopic findings considered separately and together as a total score and its correlation with cross‐sectional and predictive relationships with EPOS control status. We further sought to determine meaningful thresholds of endoscopic findings that defined future poor disease control [12].

## 2 Methods

### 2.1 Study Design and Study Population

Between 2017 and 2023, a prospective study was conducted at Northwestern Medicine involving adult patients diagnosed with CRS who underwent ESS. The study enrolled a total of 188 individuals with bilateral disease confirmed by CT (Lund–Mackay score ≥ 4), including 82 patients CRS with nasal polyps (CRSwNP) and 106 CRS without nasal polyps (CRSsNP). Patients were excluded if they showed minimal radiologic signs of inflammation, had recurrent acute rhinosinusitis, were on anticoagulation therapy or had bleeding disorders, had undergone solid organ transplantation requiring immunosuppressants, or had communicable infectious diseases such as hepatitis B or C. Baseline data, including demographics, comorbidities, medication usage, and extent of surgery [13] were collected at the time of surgery. The extent of surgery was categorized into three tiers: Tier 1, defined as limited ESS involving the maxillary and/or ethmoid sinuses, similar to a Draf I procedure; Tier 2, comprising bilateral ethmoidectomy and maxillary antrostomy with a Draf IIa/IIb frontal sinusotomy and/or sphenoidotomy; and Tier 3, representing full ESS with a Lothrop approach (Draf III) to the frontal sinuses [13]. Patients were followed at two postoperative intervals: 6–12 months (V1, short‐term) and 18–60 months (V2, long‐term). At each visit, patients completed the Sino‐Nasal Outcome Test‐22 (SNOT‐22) and 12‐item Chronic Rhinosinusitis Patient Reported Outcome (CRS‐PRO) [14], underwent an NE, and had a sinus CT scan. The prescription of systemic corticosteroids or antibiotics related to CRS was recorded via retrospective chart review. Of the 188 patients enrolled, 103 completed both V1 and V2 evaluations. This study was approved by the Institutional Review Board of Northwestern University Feinberg School of Medicine (IRB No. STU00202510).

### 2.2 Endoscopic Finding and Definition of CRS Disease Control

Assessment of disease control was retrospectively categorized into controlled, partly controlled or uncontrolled using the prospectively collected symptom items, and NE items. Medication use was retrospectively ascertained at each study visit based on prescription records. To operationalize control status defined by the European Position Paper on Rhinosinusitis and Nasal Polyps (EPOS) 2020 guidelines [1], symptom control items were assessed using corresponding items on the SNOT‐22 questionnaire that aligned with EPOS domains (nasal blockage, rhinorrhea/postnasal drip, facial pain/pressure, smell disturbance, and sleep disturbance/fatigue). An individual item score of ≥ 3 was indicative of bothersome symptoms [15]. “Controlled CRS” was defined as all respective SNOT‐22 symptom scores ≤ 2, with no use of systemic corticosteroids or antibiotics within the preceding six months as described previously [1]. Patients who did not meet these criteria were classified as having “suboptimal CRS control”, encompassing both partly controlled and uncontrolled cases in EPOS2020 [1].

NE findings were documented using the MLK scoring system, which evaluates edema, discharge, and polyps on a scale from 0 to 2 with a maximum total score of 12 [4]. Each nasal cavity was evaluated independently. The added value of endoscopic findings was studied in relation to concurrent and future disease control. For clarity, we represent a definition of CRS control^−NE^ in reference to control status defined without endoscopic results and CRS control^+NE^ when endoscopic results were incorporated as espoused in the EPOS2020.

### 2.3 Statistical Analysis

Statistical analyses were performed using R version 4.4.1(R Foundation, Vienna, Austria) and Prism GraphPad software version 10 (La Jolla, CA). Sankey diagrams were used to illustrate transitions in CRS control status between V1 and V2 visits. Categorical variables were expressed as counts and percentages, while continuous variables were summarized using mean and standard deviations (SDs) or median and interquartile range (IQR). The chi‐square test was used for comparing categorical variables, and the independent *t*‐test or Mann–Whitney *U* test was used for continuous variables. A receiver operating characteristic (ROC) curve analysis was performed using endoscopic findings (individual MLK subscores, total MLK score, and various MLK thresholds combined with V1 control status) to evaluate their ability to predict CRS control status. The area under the curve (AUC) was calculated for each predictor, with values closer to 1.0 indicating higher accuracy of prediction. Area under the ROC curve (AUC) values were interpreted as follows: < 0.7 indicated poor performance, 0.7–0.8 fair, 0.8–0.9 excellent, and > 0.9 outstanding. Other performance metrics, including the AUC with 95% confidence intervals, were calculated. A two‐tailed *p*‐value of < 0.05 was considered statistically significant for the aforementioned analyses.

## 3 Results

### 3.1 Patient Characteristics

The study enrolled 188 adult patients with a mean age of 46.3 years, comprising individuals with nasal polyps (CRSwNP, *n* = 82) and without nasal polyps (CRSsNP, *n* = 106). Detailed demographic and clinical characteristics are presented in Table 1. Note that 103 patients completed both the V1 and V2 visits. There were no significant differences in age, sex, and comorbidity status among patients who completed only V1 compared to those completing both visits (not shown).

**TABLE 1:** Patient characteristics and concurrent outcome measures of CRS controlled and poorly controlled status at different visits: (A) Short‐term visit (V1). (B) Long‐term visit (V2).

(A) Short‐term visit (V1)			
At V1	Controlled^−NE^	Suboptimal control^−NE^	*p*‐value
Number (%)	79	109	
**Demographic**			
Age (years), mean (SD)	43.6 (13.7)	48.3 (14.7)	0.02^*^
Sex (female)	39 (49.4%)	60 (55%)	0.53
CRSwNP	39 (49.4%)	43 (39.4%)	0.23
**Comorbidity**			
Allergic rhinitis	49 (62%)	57 (52.3%)	0.24
Asthma	34 (43%)	60 (55.0%)	0.14
AERD	3 (3.8%)	7 (6.4%)	0.52
Prior ESS	24 (30.4%)	47 (43.1%)	0.10
Former/current smoker	14 (17.7%)	29 (26.6%)	0.21
Preoperative visit results			
SNOT‐22, mean (SD)	43.63 (20.15)	49.01 (19.11)	0.11
CRS‐PRO, mean (SD)	24 (8.87)	27.06 (8.59)	0.48
LM score, mean (SD)	12.32 (5.23)	11.69 (5.39)	0.46
**Surgical tier**			
Tier 1 (M+E only)	10 (12.7)	19 (17.4)	0.47
Tier 2	63 (79.7)	73 (67)	0.07
Tier 3 (Draf III)	6 (7.6)	17 (15.6)	0.15
**V1 visit results**			
SNOT‐22, mean (SD)	9.75 (8.68)	29 (17.9)	< 0.0001^****^
CRS‐PRO, mean (SD)	6.57 (5.03)	17.46 (9.67)	< 0.0001^****^
LM score, mean (SD)	3.57 (3.46)	6.42 (4.86)	< 0.0001^****^
Rescue medication usage (steroid/antibiotics)	0 (0%)	43 (39.4%)	< 0.0001^****^
**V1 endoscopic scores (mean)**			
MLK edema	0.52	1.04	0.003^**^
MLK discharge	0.59	1.02	0.046^*^
MLK polyp	0.15	0.38	0.07
Total MLK	1.27	2.43	0.002^**^

(B) Long‐term visit (V2)			
At V2	Controlled^−NE^	Suboptimal control^−NE^	*p*‐value
Number (%)	45	58	
**Demographic**			
Age (years), mean (SD)	46.9 (12.2)	49.0 (19.1)	1
Sex (female)	22 (48.9%)	31 (53.4%)	0.8
CRSwNP	23 (51.1%)	20 (34.5%)	0.14
**Comorbidity**			
Allergic rhinitis	24 (53.3%)	38 (65.5%)	0.3
Asthma	21 (46.7%)	37 (63.8%)	0.12
AERD	2 (4.4%)	3 (5.2%)	1
Prior ESS	15 (33.3%)	24 (41.4%)	0.53
Former/current smoker	12 (26.7%)	16 (27.6%)	1
Preoperative visit results			
SNOT‐22, mean (SD)	40.06 (20.91)	49.14 (19.36)	0.06
CRS‐PRO, mean (SD)	22.93 (9.82)	26.58 (7.3)	0.13
LM score, mean (SD)	11.4 (5.31)	11.6 (5.49)	0.76
**Surgical tier**			
Tier 1 (M+E only)	10 (22.2)	9 (15.5)	0.53
Tier 2	31 (68.9)	38 (65.5)	0.8
Tier 3 (Draf III)	4 (8.9)	11 (19)	0.24
**V2 visit results**			
SNOT‐22, mean (SD)	8.44 (7.09)	31.88 (16.11)	< 0.0001^****^
CRS‐PRO, mean (SD)	6.27 (4.65)	18.98 (8.66)	< 0.0001^****^
LM score, mean (SD)	4.73 (3.77)	5.48 (4.51)	0.53
Rescue medication usage (steroid/antibiotics)	0 (0%)	9 (15.5%)	0.005^**^
**V2 endoscopic scores (mean)**			
MLK edema	0.6	0.93	0.13
MLK discharge	0.49	1.09	0.02^*^
MLK polyp	0.22	0.29	0.35
Total MLK	1.31	2.31	0.03^*^

Abbreviations: M+E only, maxillary and/or ethmoid sinus surgery only; ^−NE^, CRS control status is defined by symptoms and medication use, excluding nasal endoscopic (NE) findings.

^∗^ *p* < 0.05.

^∗∗^ *p* < 0.01.

^∗∗∗^ *p* < 0.001.

^∗∗∗∗^ *p* < 0.0001.

### 3.2 CRS Control Status Transition From V1 to V2 and Its Association With Endoscopic Findings

**FIGURE 1:**
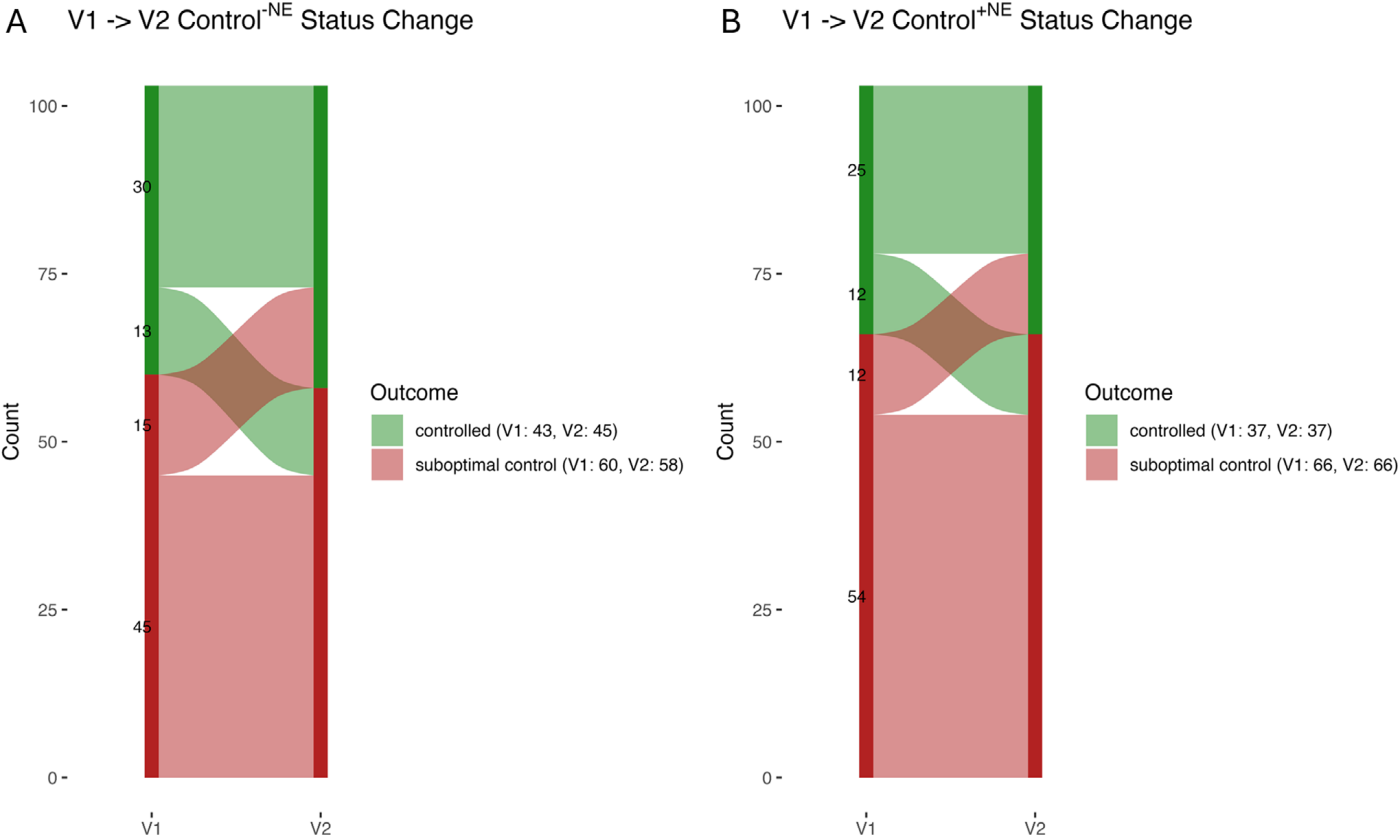
Sankey plot of CRS control status transition from V1 to V2. (A) CRS control^−NE^ status. (B) CRS control^+NE^ status. Six of 103 (5.8%) patients at V1 and 8 of 103 (7.8%) patients at V2 changed their control status after incorporating endoscopic findings. During the follow‐up period, 13 of 43 patients (30.2%) in the CRS control^–NE^ status group and 12 of 37 patients (32.4%) in the CRS control^+NE^ status group experienced disease progression. V1, 6–12 months after ESS; V2, 18–60 months after ESS; ^−NE^, CRS control status is defined by symptoms and medication use, excluding nasal endoscopic (NE) findings; ^+NE^, CRS control status is defined by symptoms, medication use and nasal endoscopic (NE) findings (with MLK ≥ 3 as the threshold).

Sankey plots were utilized to visualize the transitions in control^−NE^ status between the V1 and V2 visits (Figure 1). A total of 30 out of 43 patients (69.8%) in the control^−NE^ and 45 out of 60 patients (75%) in the suboptimal control^−NE^ group maintained the same level of disease control in V1 and V2. When applying MLK ≥ 3 as the threshold (subsequent analysis provided) to define diseased mucosa, 25 of 37 patients (67.6%) in the controlled^+NE^ group and 54 of 66 patients (81.8%) in the suboptimal control^+NE^ group maintained the same level of disease control.

**FIGURE 2:**
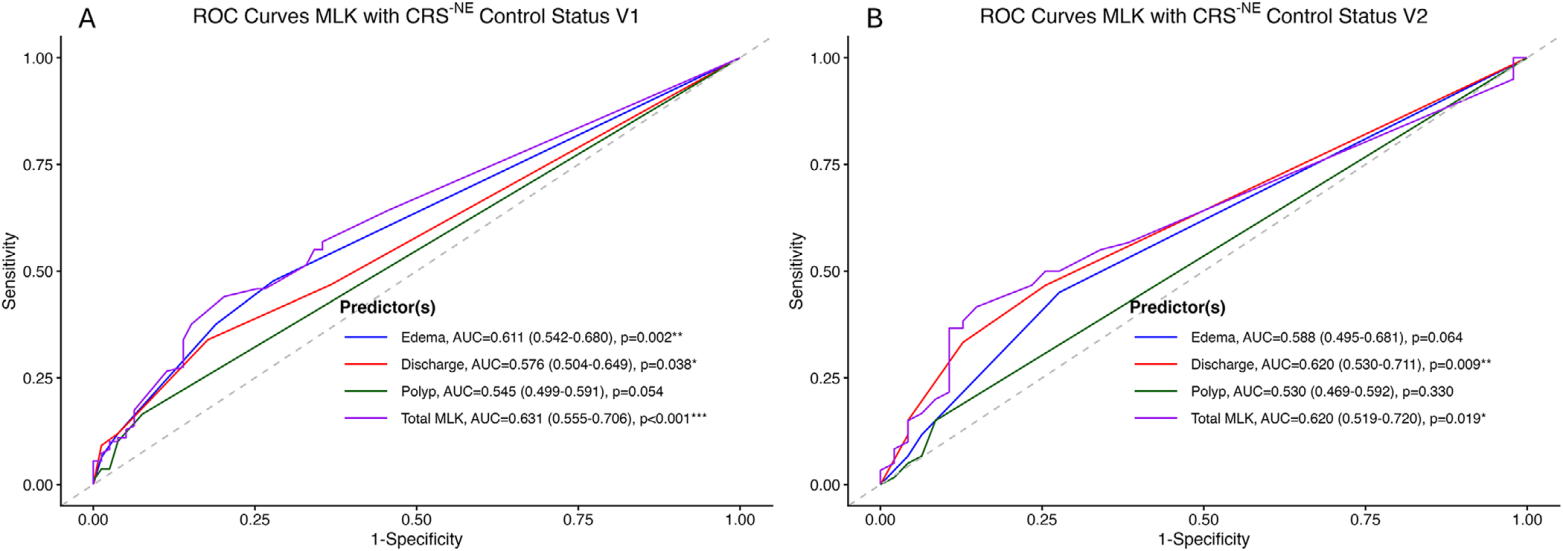
ROC curve analysis for predicting concurrent CRS control^−NE^ status. **(A)** Short‐term visit (V1, 6–12 months after ESS). **(B)** Long‐term visit (V2, 18–60 months after ESS). When assessing CRS control^−NE^ status with concurrent nasal endoscopy findings, the total MLK score demonstrated the highest correlation at both V1 and V2 timepoints (AUC = 0.631 at V1; AUC = 0.620 at V2), although the overall correlation was poor. ROC, receiver operating characteristic; ^−NE^, CRS control status is defined by symptoms and medication use, excluding nasal endoscopic (NE) findings; MLK, modified Lund–Kennedy endoscopic score; AUC, area under the curve. ∗*p* < 0.05; ∗∗*p* < 0.01; ∗∗∗*p* < 0.001; ∗∗∗∗*p* < 0.0001.

To assess the relationship between endoscopic findings and concurrent CRS disease control^−NE^, we analyzed correlations between component and total MLK scores and control^−NE^ status at each visit. Endoscopic findings showed poor, but significant association, with concurrent CRS control^−NE^ status; total MLK scores demonstrated the highest AUC values of 0.631 (0.555–0.706) for V1 (*p* < 0.001) and 0.62 (0.519–0.720) for V2 (*p* < 0.05), respectively, for determining concurrent control^−NE^ (Figure 2A,B). The analyses of individual MLK components (edema, discharge, and polyp scores) found that no individual component outperformed the total MLK score (AUC range 0.555–0.706 at V1; 0.519–0.720 at V2), although discharge severity demonstrated a significant association with control^−NE^ status whereas polyp size did not.

### 3.3 Endoscopic Findings and CRS Control Status Prediction

**FIGURE 3:**
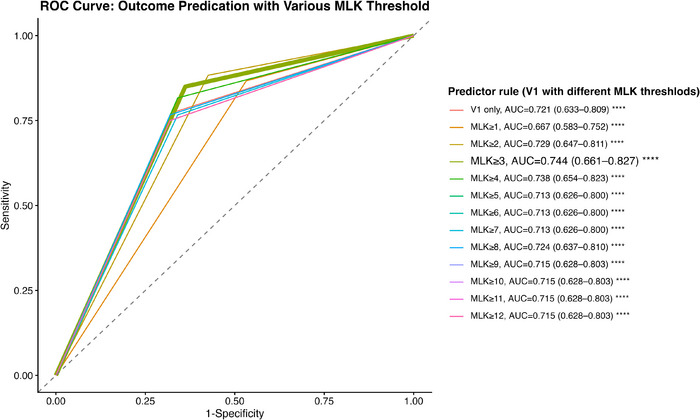
Comparison of ROC curves for long‐term CRS control^−NE^ status prediction (18–60 months after ESS). When using V1 control status, with or without nasal endoscopy findings, to predict V2 control^−NE^ status, the MLK score with a threshold of ≥ 3 demonstrated the highest predictive accuracy (AUC = 0.744, *p* < 0.0001****). ^−NE^, CRS control status is defined by symptoms and medication use, excluding nasal endoscopic (NE) findings; V1, 6–12 months after ESS; ROC, receiver operating characteristic; MLK, modified Lund–Kennedy endoscopic score; AUC, area under the curve. ∗*p* < 0.05; ∗∗*p* < 0.01; ∗∗∗*p* < 0.001; ∗∗∗∗*p* < 0.0001.

Next, we examined whether incorporating endoscopic findings (MLK scores) enhanced the *prediction* for CRS control^−NE^ status at V2. Every possible MLK total score threshold was utilized to define a V1 control^+NE^ status. An MLK ≥ 3 offered the highest predictive accuracy for V2 control status compared to all other MLK thresholds (AUC: 0.744 [0.661–0.827], *p* < 0.0001) (Figure 3). Similarly, using MLK ≥ 3 as a categorical variable without utilizing symptoms was also significantly associated with V2 control status (chi‐square test, *p* < 0.05). Despite the modest effect, it provided a significant improvement in predicting long‐term V2 control^−NE^ status.

## 4 Discussion

Our study investigated the stability of EPOS definitions of CRS disease control and the effect of considering the optional endoscopic findings on control status definition and stability. Endoscopic assessments were poorly associated with concurrent CRS control. We found the composite total MLK score had the strongest association with *concurrent* disease control outperforming all its individual subcomponents. Despite poor association with concurrent control, the addition of endoscopic findings did marginally enhance prediction of *future* CRS control compared to control defined without endoscopy. Notably, in our cohort, an MLK score threshold of 3 was identified as critical: patients with an MLK score ≥ 3 appear and concurrent control group were more likely to progress to suboptimal control status at the long‐term outcome (2–5 years of postoperative follow‐up) assessment, suggesting MLK ≥ 3 as an appropriate threshold for “diseased mucosa.”

Disease control is an important outcome measure and has been widely discussed [16, 17, 18]. According to the EPOS 2020 [1], CRS disease control is classified as controlled, partly controlled, or uncontrolled. This classification is based on clinical symptoms, the need for rescue treatment—supplemented by NE findings when available. The presence of at least one criterion indicates partly controlled, while meeting three or more criteria signifies an uncontrolled state. The EPOS 2020 definition of disease control has been widely applied in clinical research, including biologics or surgery outcome evaluation [16, 18, 19]. Patients with suboptimal control disease trended toward having more severe pre‐ESS baseline symptom burden, and Draf III procedures were more frequently associated subsequent suboptimal control in our study. Some validation studies evaluating the EPOS 2012 criteria have suggested that they may overestimate the proportion of uncontrolled patients compared to patient self‐perception [7, 8, 20], which led to modifications in the EPOS 2020 CRS criteria [1]. However, the definition of endoscopic findings remained unclear. In EPOS 2020, the nasal polyps, purulent secretions, and inflamed mucosa are listed as indicators of diseased mucosa despite their qualitative differences [1]. No clear thresholds have been established or supported by real‐world data.

For rhinologists, endoscopy serves as a valuable assessment tool with positive findings potentially leading to treatment escalation [3, 9]. There are many NE criteria being used, including NPS, Lund–Kennedy score (polyp, edema, discharge, crusting, and scarring), MLK score (polyp, edema, and discharge) and discharge, inflammation, and polyps/edema (DIP) endoscopic scoring system, although these various scoring systems frequently focus on similar features [4, 5, 21]. The strength of the correlation between endoscopic findings and patient‐reported outcome is usually poor [5, 7, 9, 21, 22] and rarely changes CRS control status evaluation using current EPOS definitions [8] (4.9% in previous study [8] and 5.8%–7.8% in our study). Ryan et al. reported a lack of correlation between endoscopic findings and patient symptoms post‐ESS [23]. A recent systematic review indicated poor correlation between nasal polyp grading systems and patient‐reported outcome measures (PROMs) such as SNOT‐22 [6]. In contrast, Psaltis et al. found correlations between concurrent MLK scores and SNOT‐22 subscores [4], similar to our findings. Although significantly correlated with current control status, we found that the prediction of concurrent control using total MLK score alone was inaccurate (AUC = 0.631 at V1; AUC = 0.620 at V2) though statistically significant. Nonetheless, adding endoscopic features seemed more useful for improving *prediction* of long‐term control status (AUC = 0.721 when excluding NE; AUC = 0.744 when incorporating NE). This result echoes findings we recently published where concurrent symptomatic measures are poorly related to radiographic severity, but achievement of radiographic normalization did slightly improve long‐term symptom control [10].

The endoscopic definition of “diseased mucosa” has not been well defined, though prior studies suggest endoscopy findings have a role in treating CRS [1, 3, 24]. A recent expert consensus suggested an MLK ≥ 4 threshold for treatment escalation based solely on expert opinion [25]. Our results now first demonstrate that a lower threshold (total MLK score ≥3), combined with current control status, improves the predictability of long‐term CRS outcomes (AUC: 0.744, *p* < 0.0001; chi‐square test, *p* < 0.05). We additionally analyzed each of the individual components of the MLK score and found none outperformed the total score, although discharge and edema consistently outperformed polyp size in its relationship to concurrent disease control at both time points. These findings validate recent recommendations that patients should be assessed symptomatically and objectively after 3–12 months sinus surgery to tailor treatment and ensure resolution of inflammation post‐ESS.

However, this study has several limitations. First, the relatively small sample size of 103 patients with complete follow‐up limits our ability to perform a detailed analysis of what defines diseased mucosa. Although we found that MLK ≥ 3 may serve as a predictor of long‐term outcomes, further granularity—such as whether discharge contributes more significantly than other components—will require evaluation in a larger dataset. Second, the incremental predictive value provided by endoscopic findings, while significant, remains modest. Additionally, our findings are limited to postoperative CRS patients, and their applicability to patients managed exclusively with medical therapy remains uncertain. As a single‐center study, subjective variability across assessors endoscopic scoring may exist, although a validated endoscopic scoring system was utilized. Multicenter and larger prospective studies are needed to confirm our preliminary findings.

## 5 Conclusion

Integrating NE findings with current disease control status enhances the prediction of future CRS control status [26]. A combined score of ≥ 3 appears to be a useful threshold. These findings support the routine incorporation of NE into follow‐up assessments as part of a more comprehensive CRS evaluation. Furthermore, the use of the MLK score may assist in guiding treatment escalation, even in asymptomatic patients. An MLK score of ≥ 3 may serve as a potential indicator of poorer long‐term disease control. This research provides novel, real‐world evidence supporting the predictive value and suggested threshold of endoscopic findings in assessing CRS disease control.

## Funding

This work was supported by NIH grants R01 AI134952, R01 DC016645, and the Chronic Rhinosinusitis Integrative Studies Program 2 (CRISP2) (P01 AI145818); and grants from the National Taiwan University Hospital, Yunlin Branch (NTUHYL114S008 and NTUHYL113N002).

## Conflicts of Interest

D. B. Conley reports personal fees for Intersect ENT and XORAN. R. C. Kern reports personal fees from Sanofi, Novartis, Lyra Pharmaceutical, and Neurent. A. K. has received a research support from Regeneron‐Sanofi. B. K. Tan reports personal fees and grant support from Sanofi Regeneron/Genzyme. The rest of the authors declare that they have no relevant conflicts of interest.
